# Farming System for Nutrition-a pathway to dietary diversity: Evidence from India

**DOI:** 10.1371/journal.pone.0248698

**Published:** 2021-03-18

**Authors:** Aliza Pradhan, Raju S., Nithya D. J., Akshaya Kumar Panda, Rupal D. Wagh, Mahesh R. Maske, Bhavani R. V.

**Affiliations:** 1 ICAR-National Institute of Abiotic Stress Management, Baramati, Pune, India; 2 M. S. Swaminathan Research Foundation (MSSRF), Taramani, Chennai, India; 3 Independent Researcher, Amravati, India; 4 Borlaug Institute for South Asia (BISA), New Delhi, India; Michigan State University, UNITED STATES

## Abstract

Farming is the main livelihood of a majority of people in India. The country is also home to a large population of undernourished people. This indicates potential for mainstreaming the nutrition dimension in the farming system to impact on nutrition outcomes. A Farming System for Nutrition (FSN) study was conducted in two agro-ecologically different locations from 2013–2018, to explore the feasibility of nutrition-sensitive agricultural interventions. The baseline survey in 2013–2014 revealed that the population in the study area was largely undernourished and that household diets were cereal-dominated. The FSN model was designed in consultation with community members, to increase availability of nutrient-dense cereals and pulses, by enhancing production and crop diversification at the farm level, promoting cultivation of nutrient-rich fruits and vegetables in nutrition gardens and supporting interventions to promote access to animal foods. Nutrition awareness initiatives were undertaken to build capacity at the local level and translate production diversity to consumption diversity. An endline survey was conducted in 2017 (July-October), following three years of intervention. Crop, vegetable and animal food production and food consumption was compared with the baseline data. There was evidence of higher production and consumption of nutrient rich foods, improved household dietary diversity; and understanding and acceptance of nutrition-sensitive agriculture. The number of items consumed under each food group, frequency of consumption of food and average per capita intake of nutrient-rich foods were found to have improved. The results provide evidence regarding feasibility of location-specific FSN models to promote sustainable and healthy diets, using locally available plant and animal food resources, to address nutrition deficiencies in farm families.

## Introduction

Agricultural research and related policies in developing countries like India have largely focused on building a food environment that can assure staple food production and availability for a growing population. This has led to an important concern, particularly for those in the low-income groups as they are consuming mainly staples, which are high in carbohydrates, but low in micronutrients [1,2]. Staple food items could increase energy availability, but will not improve nutritional outcomes unless consumed in conjunction with micronutrient rich foods [3–5]. This is evident from the National Family Health Survey (NFHS-4) in India, 2015–16, which revealed that forty one per cent children below five years of age, are stunted (low height for age), thirty eight per cent are underweight (low weight for height) while fifty nine per cent are anaemic. Further, around fifty four per cent women between age of 15–49 years are anaemic [6]. A majority of India’s population is dependent on agriculture as their primary source of livelihood. In a context, where a significant section of the population is malnourished and dependent on agriculture, a pathway for addressing food and nutrition security by leveraging nutrition-sensitive agriculture would have great potential. Six potential pathways through which agriculture may affect nutrition outcomes are cited in literature, with agriculture as a source of food listed as a direct pathway [7–11]. Nutrition-sensitive agricultural interventions can be a practical and sustainable way of alleviating nutritional deficiencies by targeting improved production diversity, integrating nutrient-rich foods for increased availability, consumption and better dietary diversity at the household level [12–15]. There are also several reviews assessing the evidence between nutrition-sensitive agriculture interventions and nutritional outcomes [7,16–22]. Overall, the evidence is that nutrition-sensitive agriculture programs that promote production diversity, micronutrient rich crops (including biofortified crops), dairy, or small animal rearing, can improve dietary diversity at the household level. Ruel et al. [21] concluded a systematic review of over 40 studies since 2014 by saying, “Agriculture should focus on improving dietary diversity and high-quality diets as a precursor to better nutrition outcomes”. Also, studies have shown that recognizing women’s contribution to both agricultural production and domestic reproduction, and supporting them adequately, is central to improving nutritional outcomes [23,24].

The conventional farming systems largely aim at food security with a major focus on productivity, profitability, sustainability and stability. However, increased food production and/or increased income by itself do very little towards ensuring a balanced diet for rural households [18,25]. Nutrition security therefore has to be addressed by both availability and accessibility of nutrient-rich foods at the household level, which is central to Farming System for Nutrition (FSN). It is a nutrition-sensitive agriculture approach that entails ‘the introduction of agricultural remedies to the nutritional maladies prevailing in an area through mainstreaming nutritional criteria in the selection of the components of a farming system involving crops/plants, farm animals and wherever feasible, fish’ [26]. A feasibility study on the FSN approach was undertaken in India from 2013–14 to 2017–18, to address nutrition security through improved household dietary diversity, under the research programme on Leveraging Agriculture for Nutrition in South Asia (LANSA). This paper examines the outcome of the study, i.e., changes in crop production following the FSN intervention and the impact on household consumption and dietary diversity.

## Materials and methods

### Study sites

The study was undertaken in two agro-ecologically different regions; in Koraput district of Odisha state and in Wardha district of Maharashtra state in India. The study locations were purposively selected due to their characteristic contrast with regard to agro-climatic conditions, landholding pattern, and farming practices. A detailed baseline survey of all households was undertaken in 2013–2014 at both study sites, to capture information on demographic and socio-economic characteristics, nutrition status of the population, and household food consumption pattern. Focus Group Discussions with 10–15 participants each, were conducted with groups of women and adolescent girls in all study villages, to assess the level of nutrition awareness. The discussion was conducted with women, as they are “responsible for household food preparation and child care”, and also keeping in mind the importance of nutrition status of adolescent girls as future mothers. The baseline was done across 19 villages (8 in Wardha and 11 in Koraput). The baseline survey revealed that majority (46 per cent) of people in the selected villages belonged to Scheduled Tribe (ST) community (indigenous people recognized by the government and among the most economically underprivileged and nutritionally vulnerable). The main occupation of the population was agriculture. Based on the size of operational landholding, a majority in Koraput were marginal farmers with less than one hectare of land (81 per cent) and 17 per cent were landless, while in Wardha, 36 per cent were small and marginal farmers, 19 per cent had between 2 to 4 hectares of land, and 37 per cent were landless. Agricultural wage labor was the secondary occupation. Detailed information of the study locations and baseline socio-economic status, nutrition and agriculture situation summarized herein can be found in Bhaskar et al. [27] and Nithya et al. [28].

The villages in Koraput, experience average annual rainfall of 1320 to 1520 mm, and are characterized by paddy-based subsistence cropping system. *Kharif* (June to October) is the main cropping season and the cropping pattern in the area is primarily based on land type–upland, medium land and lowland (upland indicates lands of higher elevation where there is no retention of water 24 hours after rainfall; medium land indicates levelled fields and lowland refers to lands at lower elevation where water stagnation is commonly seen even 24 hours after rainfall). In *kharif*, paddy is grown in all the three types of land while cultivation of finger millet/little millet/maize is confined to uplands only. In few medium and low land paddy fallows, subject to moisture/irrigation availability, vegetables are grown in *rabi* season (November to February) and in pre-summer, pulses (black gram and green gram) are grown. Villages in Wardha experience average annual rainfall of 807 to 1152 mm and are characterised by cotton or soybean-based commercial cropping system (commercial cropping system indicates growing of food/non-food crops primarily for sale whereas under subsistence farming, production is primarily for home consumption). In *kharif*, largely sole cropping of cotton/soybean or intercropping of these two crops with pigeon pea is seen in both irrigated and rainfed lands. In *rabi*, cultivation of wheat and bengal gram is done in irrigated condition; few farmers grow bengal gram in rainfed condition as well.

Based on logistic considerations and availability of funds, a cluster of five villages in Wardha (556 households with a population of 2254) and seven in Koraput (658 households with a population of 2845) were purposively selected for interventions under the study.

#### Disconnect between agriculture and nutrition in study locations

The majority of households in both the study locations depend on agriculture as the main source of food and income [27]. The level of under-nutrition across all age groups is high; around 40 per cent of children below five years are underweight, 35 per cent are stunted and 27 per cent wasted while 39 percent of men and 48 per cent of women have chronic energy deficiency. Anaemia levels of 60 per cent and above in children (6–59 months) and women (18 to 45 years) were reported in both locations [28]. The household diet was found to be cereal dominated with consumption of all other food groups being less than the recommended levels, indicating low dietary diversity. Although agriculture was the primary source of food and income, production practices were limited to few crops, contributing to cereal dominated food consumption pattern and reflecting disconnect between agriculture and nutrition.

### Farming System for Nutrition (FSN)

#### FSN design

The central objective was to study the feasibility of a location-specific FSN approach to improve household dietary diversity.

The survey findings were shared with the village community and farming system interventions were laid out in discussion with them. The support and advice of researchers, extension agents, subject matter specialists from universities, and research institutes in each region was also taken. A brief summary of research findings from baseline survey, the key gaps identified from both the survey and expert consultations, and the design of FSN interventions to address them, is discussed below.

1. Less area under millet cultivation and low productivity of the crop: Tribal households in Koraput traditionally consume finger millet on a regular basis but were found to be sourcing it from the market. In Wardha, the land under sorghum cultivation was found to have been replaced by commercial crops. The FSN design focused on increasing area and productivity of both these nutrient-dense millet crops.
2. Low availability of pulses: There was no pulse crop during the *kharif* season in Koraput and productivity was low during *rabi*. In Wardha, productivity was low and there was limited diversity. The consumption of pulses was below recommended levels in both locations. Emphasis was given to increase area under cultivation, improve productivity and increase diversity in pulse crops cultivated.
3. Low cultivation and consumption of all groups of vegetables and fruits: Nutrition gardens of fruits and vegetables were promoted in both locations with a seasonal calendar of all three groups of vegetables that could be grown. Among tubers, beta carotene-rich Orange-Fleshed Sweet Potato (OFSP) in particular, was promoted.
4. Low consumption of animal proteins: This was below recommended levels with consumption largely during special occasions and festivals. Fishery in Koraput and backyard poultry in Wardha was promoted to increase access to animal sources of protein and micronutrients.
5. Knowledge gap: Nutrition awareness was made an integral part of the design with focus on understanding of balanced diet and creating awareness on the nutrient value of different plant/animal foods.

**Fig 1:**
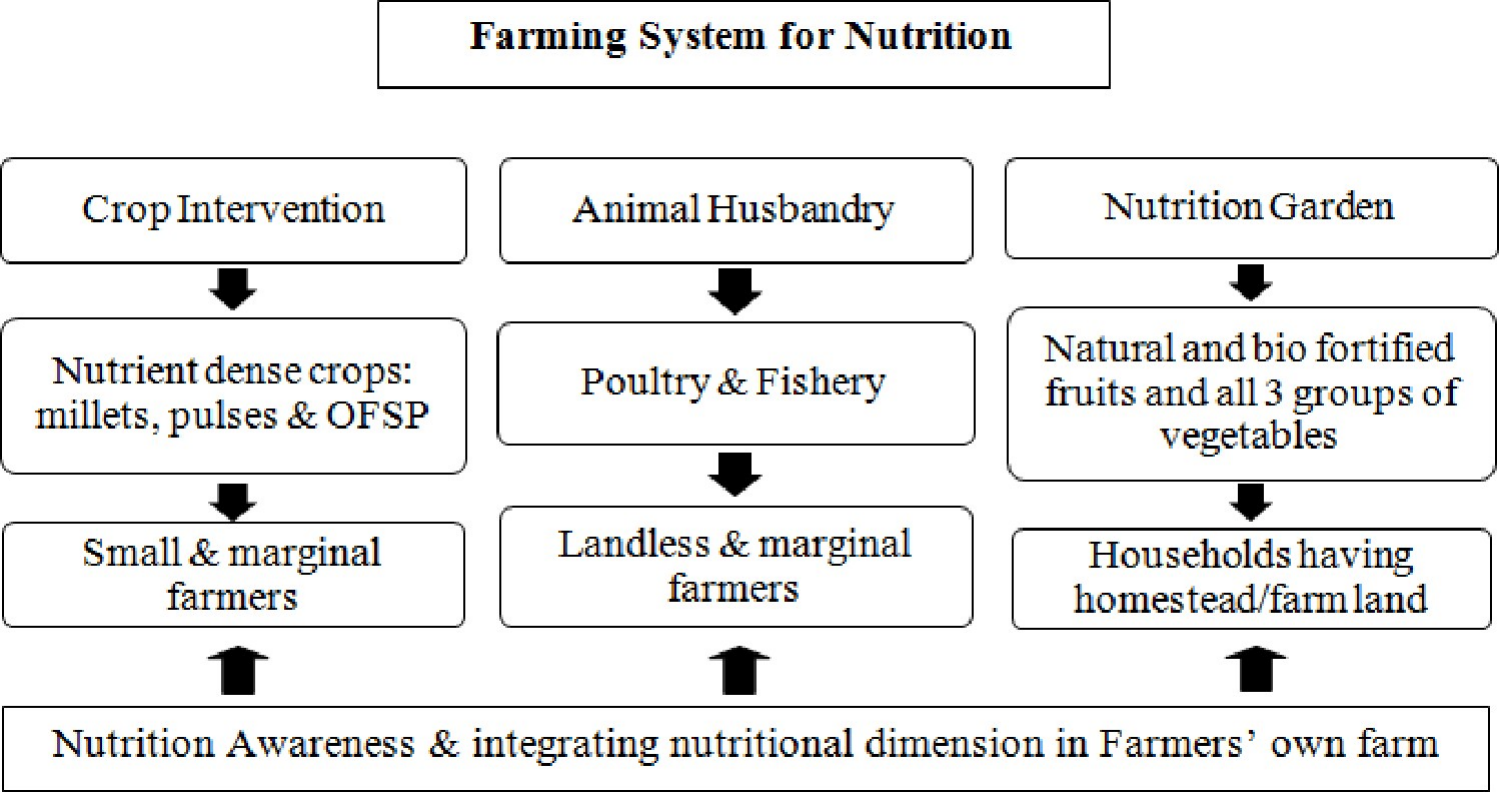
Farming System for Nutrition interventions.

In sum, the FSN design focused on, on-farm crop interventions, promotion of nutrition gardens, increasing access to animal food sources, and nutrition awareness. This is schematically presented in Fig 1 and discussed in detail in the subsequent paragraphs.

#### Crop interventions

Enhancing production and productivity of nutrient-dense crops through increase in area, varietal substitution, and crop diversification were the major crop related strategies adopted in the study area.

In the first two years. i.e., from late 2013 to 2014–2015, with the baseline survey underway, On-Farm Demonstrations (OFDs) were undertaken, mainly to build rapport with the farm households. A number of technologies and practices already developed and recommended by agricultural universities in respective states as a part of their research programs, were piloted initially, in fields of a few interested farm households. This also served as a means for farmers to see their performance in comparison with the practices being followed by them. The OFDs considered each farmer’s plot as a replication and the allotted land area was split into known portions to compare farmers’ practice with recommended agronomic practice. Men and women farmers were involved in the design, implementation and assessment stages; this served as a sensitization and training phase. On-site field staffs were assigned village-wise responsibility to facilitate and monitor implementation of FSN interventions ensuring uniformity regarding date of sowing, plant spacing, varieties, input use, harvesting etc. They were in turn monitored and directed by a project coordinator.

Performance evaluation of identified crop interventions was done on the basis of crop productivity, economics of crop production and farmer experience. Crop-specific yield attributing parameters as well as total yield under FSN crop intervention were measured and compared with those under traditional farm practices. The cost of cultivation was calculated by taking input costs such as seed, fertilizer and hired labour; total return was calculated by multiplying yield with the average market price of the respective crop. The net return was calculated by subtracting the total cost of cultivation from the total return. Details of different crop interventions are reported in Pradhan et al. [29,30] and S1 Table.

Based on the comparative assessment of OFDs over two years, and feedback from farmers and technical experts, core crop interventions were finalized for each study site.

#### Nutrition garden

It was seen from the baseline survey that 30 per cent of the 658 households in the seven villages in Koraput and 19 per cent of the 556 households in the five villages in Wardha, practiced traditional home gardening with limited cultivation of only one or two types of vegetables, primarily for consumption. The area of backyard land ranged from 80 to 600 sq m in Koraput and 6.3 to 15.9 sq m in Wardha. As backyard area was less in Wardha and a large part of the time was spent in the fields, many households used to grow vegetables on a patch in the field itself. Considering the minimal cultivation and consumption of vegetables and fruits, nutrition gardens were promoted in interested households in 2014–15 at both study sites. A seasonal calendar of locally available vegetables with emphasis on their nutrient content was prepared in consultation with household members, Integrated Child Development Service Scheme (ICDS) and ASHA (Accredited Social Health Activist) workers and nutritionists. Seed kits comprising seeds of seasonal vegetables from all three vegetable groups viz., green leafy vegetables, roots/tubers and other vegetables along with saplings of naturally fortified fruits and tree species (e.g. moringa, lemon, amla, papaya, guava, mango) were distributed to households with a backyard area. Awareness programmes, trainings and workshops were organized in each village at both project sites with the help of staff members and subject matter experts. An annual calendar containing pictures for each month selected from a drawing competition conducted in the village schools was created. This was prepared with related nutrition messages and distributed to all households in the study villages. Exposure visits, lectures on specific topics for selected groups like pregnant and lactating women and adolescent girls on dietary requirements, exhibition on pulses and food groups and demonstrations showing preparation of recipes, seed collection and conservation were conducted regularly. Data about area, plants grown in the nutrition garden, number of harvests, yield and utilization patterns was collected with a ‘nutrition garden utilization card’. The data was collected once a week by trained village volunteers and project staff. Details on nutrition garden interventions are available in Pradhan et al. [31].

#### Animal husbandry

Interventions on animal foods were initiated considering resource availability, accessibility and socio-cultural sensitivity of the study sites. Backyard poultry was promoted among landless and marginal farmer households in Wardha; in Koraput, fishery was promoted in individual farm ponds, farmer-group managed ponds and village community ponds.

#### Nutrition awareness

Nutrition awareness programmes were conducted at individual and household levels, to enable farmers to appreciate the importance of dietary diversity and the nutrient content in different crops. These included, Focus Group Discussions on issues like anaemia and young child feeding, observance of important days like World Toilet day and Hand wash day, lectures on balanced diet and WASH. Men and women were selected from the study villages and trained on aspects of good nutrition and linking agriculture to nutrition through a learner-centred adult nutrition literacy action research programme called Community Hunger Fighters (CHF). Twenty five community members (13 male and 12 female in the age group of 25 to 48 years) in Koraput and fifteen (seven male and eight female in the age group of 18 to 37 years) in Wardha, were trained through two three-day residential training programmes on basics of nutrition and linking agriculture to nutrition. The training was provided by an expert in community nutrition assisted by project field staff, who had been oriented earlier. The CHF approach and activities, from selection of participants, training as well as spread of awareness under the action research programme has been discussed in Narayanan et al. [32]. The premise was that through such participatory action, they would be capacitated as resource persons to generate awareness on nutrition-sensitive agriculture at the ground level.

### FSN interventions

The interventions promoted are discussed in detail separately for each location, in this section.

#### Koraput

In Koraput, land is generally left fallow after the *kharif* harvest; some farmers with low/medium land with irrigation facility cultivate vegetables in *rabi* and green gram and black gram during pre-summer i.e. February to mid-April/May. Details of the FSN intervention in Koraput are given in Table 1. Suitable short-duration improved varieties of pulses with timely crop management practices were introduced to increase pulse production. Interventions were promoted in both low and medium land paddy ecosystems, to increase the cropping intensity to 200 per cent by introducing rice fallow crops such as green gram/black gram through relay cropping. Farmers who did not have upland to grow finger millet in *kharif*, were encouraged to grow a short duration finger millet variety ‘Bhairabi’ in *rabi* in paddy fallows, if irrigation facility was available. In uplands, line transplanting of an improved variety of finger millet, ‘GPU-67’ was promoted instead of broadcasting of low quality seeds of local varieties, or perennial plantations such as eucalyptus. Intercropping of an improved variety of pigeon pea ‘NTL 724’ with maize in 1:1 ratio was promoted in place of cultivation of broadcasting local varieties of maize. In upland areas where farmers usually cultivate local white flesh sweet potato, they were encouraged to grow OFSP variety ‘Kamala Sundari’.

**Table 1:** Details of FSN interventions in Koraput.

Components of FSN	Details	Cropping system	
		*Kharif*	*Rabi*
Crop	^c^Low land and medium land	Paddy	^a^Black gram/green gram
		Paddy/vegetable	^b^Finger millet
	^c^Upland	^a^Maize + ^b^Pigeon pea	Fallow
		^a^Finger millet	Fallow
		^b^Orange flesh sweet potato	
	Homestead land	^b^Household nutrition garden	
Animal husbandry	Landless & marginal farmers	^b^ Fishery	

^a^ indicates interventions where varietal replacement in combination with improved package and practices were followed for improved production

^b^ indicates interventions introduced for nutrient-rich diversified production.

^c^Upland indicates lands of higher elevation where there is no retention of water after 24 hrs of a rainfall; medium land indicates levelled fields and lowland refers to lands at lower elevation where water stagnation is commonly seen after 24 hrs of a rainfall.

Most households were growing one or two types of vegetables in land adjacent to their homes, based on their preference. They were encouraged to make this land a nutrition garden with diversified production of fruits as well as all three types of vegetables: green leafy vegetables, roots and tubers and other vegetables. By 2016–17, out of 658 households across the seven study villages, around 79 per cent had nutrition gardens compared to 30 per cent at baseline.

Freshwater fish farming was promoted as a source of both food and additional income for small farmers and landless rural households with access to water bodies. Ponds in the study villages were mainly rain-fed and used for multiple purposes: social and domestic use, livestock, crisis irrigation and fisheries in order of priority. Regular training on a package of practices including feeding practices and importance of fish consumption was given to the community with technical support from the district Fishery Department. Composite fish farming of three major species; Catla (*Catla catla*) as surface feeder, Rohu (*Labeo rohita*), as column feeder and Mrigal (*Cirhinus mrigala*) as bottom feeder were selected in the ratio of 4:3:3. Following a participatory discussion with community members in 2013–14, fresh water fish farming was piloted in three community ponds in three villages involving 36 or five per cent households. By 2016–17, the number increased to 131 households, a fourfold increase in percentage terms with 64 ponds (56 individual, six group and two community managed) under freshwater fish farming.

#### Wardha

In Wardha, where cotton and soybean often with pigeon pea as intercrop are the main crops in *kharif*, both sole cropping and intercropping of improved varieties of pigeon pea, green gram and black gram, as well as cultivation of sorghum were encouraged. Details of FSN intervention are given in Table 2. Improved varieties of pigeon pea ‘NTL-30’and ‘PKV Tara’ were introduced. In the *rabi* season, micronutrient-dense improved varieties of wheat (rich in iron and zinc) namely, ‘AKAW-4210’, ‘NIAW-1415’ and wilt-resistant chickpea variety ‘JAKI 9218’ were promoted. Soybean, although nutrient rich is not preferred for consumption and is grown only as a commercial crop; it was therefore not promoted.

**Table 2:** Details of Farming System for Nutrition interventions in Wardha.

Components of FSN	Details	Cropping system	
		*Kharif*	*Rabi*
**Crop**	Irrigated	Intercropping of cotton or soybean with ^a^pigeon pea/^b^green gram/^b^black gram	Fallow
		Sole cropping of ^a^pigeon pea or intercropping with ^a^sorghum	Fallow
		Sole/mixed cropping of ^a^sorghum/^b^green gram/^b^black gram	^a^Wheat/^a^bengal gram
	Rainfed	Intercropping of cotton or soybean with ^a^pigeon pea/^b^green gram/^b^black gram	Fallow
		Sole cropping of ^a^pigeon pea or intercropping with ^a^sorghum	Fallow
		Sole/mixed cropping of ^a^sorghum/^b^green gram/^b^black gram	^a^Bengal gram
	Homestead land	^b^Household nutrition garden	
**Animal husbandry**	Landless/Marginal farmer	^b^Backyard poultry	

^a^ indicates interventions where varietal replacement in combination with improved package and practices were followed for improved production

^b^ indicates interventions introduced for nutrient rich diversified production.

Unlike Koraput, cultivation of vegetables in backyard area was not a usual practice in Wardha as the average backyard area was much smaller and farmers spent most of their time in fields growing labour-intensive commercial crops in *kharif*. Lack of water availability was also another factor. Therefore, farmers were encouraged to grow vegetables and fruits both in fields and in the backyard area of their homes as part of the nutrition garden intervention. By 2016–17, out of 556 households across five study villages, around 40 per cent had nutrition garden compared to 19 per cent at baseline. Households with no backyard land were also encouraged to form groups and cultivate vegetables and fruits on common land (with permission from local government body called ‘Panchayat’) in the village as ‘community nutrition garden’ and share the produce [26].

Backyard poultry was introduced to 25 landless/marginal farmers, to promote access to animal protein food. Each household was provided with 16 chicks of improved poultry breed namely *Vanaraj*, *Giriraj*, *Swarnadhara*, *Rhode Island Red* along with other critical inputs (feed, cage). Several training programmes, covering aspects of backyard poultry management, use of low-cost poultry feed, vaccination, health management were organized with technical support from State Animal Husbandry Department.

### Endline survey

An endline survey was conducted in late 2017 to assess the impact of the FSN interventions. A sample of approximately 30 per cent of households was randomly drawn from the total number of households for ensuring statistical analysis at 90 per cent confidence level; i.e., 190 households were selected in each study site. In Koraput, the sample included 156 households that participated in one or more of the interventions; the number was 158 households in Wardha. This set of intervention households was considered for analysis of changes between baseline and endline and the findings are presented in this paper.

### Compliance with ethical standards

The study methods were approved by the Ethics Committee of the Board of Trustees, M.S.Swaminathan Research Foundation. The purpose of the survey was explained to the respondent in each household by the field investigator and oral informed consent obtained before proceeding to administer the questionnaire.

### Data collection and analysis

The household surveys were conducted using well-structured questionnaires that were piloted and then finalized. The data was collected by educated youth enumerators who underwent training in conduct of survey and practiced administering the same and recording responses, before rollout. The survey team comprised seven enumerators in Koraput and six in Wardha, each led by a team leader. The process was coordinated by a survey manager supported by a nutritionist, to monitor conduct of the diet survey.

Details on agriculture were collected from the farmer or the head of the household (this was the male or female in the house responsible for taking major decisions related to farming). Total cost and production details of each intervention crop were recorded through a structured format; details on food consumption were collected from the female in the household who cooked the food. Data on food consumption pattern (quantity, frequency and source) was collected through a 30-day recall (in order to collect information on all types of foods consumed in that particular season) using semi-quantitative questionnaire. The consumption in grams per consumer unit per day was compared with the recommended dietary intake (RDI) of different foods in a day prescribed by the Indian Council of Medical Research (ICMR) [33]. Diet survey using 24-hour diet recall was done for 150 households drawn randomly from the sample households and the household dietary diversity score (HDDS) calculated. The diet survey was done continuously on all days (keeping in mind that most of the households consume animal foods during weekends) excluding festival days, function at the household/village and when there were guests visiting the households. The calculation of HDDS was based on the method given by Kennedy et al. [34]. T test and ANOVA were performed to analyse the significance difference in dietary diversity between baseline and endline using SPSS software. The data was checked for normality by visual inspection of histograms and Q-Q plots. The data on crop, vegetable and animal food production, and food consumption pattern (for the period of May-August 2017) was compared with the baseline data for the same set of households, collected during the same period in 2014. An external evaluation was commissioned to assess the impact of the CHF approach.

## Results

The changes in production and consumption are discussed separately for the two locations followed by examining the changes in dietary diversity and nutrition awareness.

### Production diversity and food consumption pattern

#### Koraput

*Production diversity*. Cultivation of improved high yielding varieties of green gram ‘SML 668’ and black gram ‘TK 94–2’ pulse varieties showed 84 and 95 per cent higher yields than the local varieties being cultivated (Table 3). In the case of finger millet, there was a six-fold increase in yield and 50 per cent of households were cultivating the crop as against 29 per cent in the baseline. Maize-pigeon pea intercropping resulted in additional yield of 953 kg ha^-1^ pigeon pea seeds along with maize from the same patch of land with cultivation by 21 per cent of households in endline as against nil at baseline. Cultivation of OFSP yielded 6300 kg ha^-1^ with cultivation by 22 per cent of households in endline. Cultivation of fruits and vegetables was a common practice and while the percentage of households with nutrition garden remained the same, the number of vegetable varieties grown had increased from just one or two vegetables during baseline, to cover different groups of vegetables. Further, at endline, 32 per cent of households were practicing fishery, with average annual production of 37 kg per 1000 m^2^ pond area, as against nil during baseline.

**Table 3:** Comparison of FSN crop interventions, cultivation and production status between Baseline and Endline, Koraput (N = 156).

Crop interventions under FSN	Baseline (2014)	Endline (2017)
Finger millet		
% of households cultivating	29	50
^a^Production (kg ha-1)	400±48	2513±28
Pigeon pea		
% of households cultivating	0	21
Production (kg ha-1)	0	953±17
OFSP		
% of households cultivating	0	22
Production (kg ha-1)	0	6300±22
Green gram		
% of households cultivating	14	37
Production (kg ha-1)	245±82	450±12
Blackgram		
% of households cultivating	5	29
Production (kg ha-1)	220±64	430±11

^a^indicates production figures in value ± standard deviation.

*Food consumption pattern*. Apart from cereals, average consumption of fruits, leafy vegetables, and other vegetables met the RDI (fruits: 87 g/CU/day at baseline to 124 g/CU/day at endline against RDI 100 g/CU/day; leafy vegetables: 57 g/CU/day to 125 g/CU/day against RDI 100 g/CU/day; other vegetables: 116g/CU/day to 245 g/CU/day against RDI 200 g/CU/day. The slight fall in consumption of meat and poultry products was offset by increase in the consumption of fishes and seafood due to fishery intervention.

Average intake of foods entirely from home production was observed to have increased, as can be seen in Table 4. This is attributable to their production on-farm and consequent availability for household consumption. For instance, the 14 per cent increase in average intake of finger millet to 80 g/CU/day from 70 g/CU/day during baseline, can be attributed to the six-fold increase in its production at farm level. The sourcing of finger millet had largely been from the market at baseline. It can also be seen from Table 3 that consumption of pigeon pea from own farm production was observed only in the endline survey, after farmers practiced intercropping of pigeon pea with maize in *Kharif* instead of the usual practice of sole cropping of maize. Diversified consumption of pulses i.e. consumption of three different types of pulses (pigeon pea, black gram, green gram) by households practicing FSN interventions was also observed. Similarly, as cultivation and consumption of sweet potato was a part of the tribal food consumption pattern, introduction of its biofortified variety i.e. OFSP (rich in Vitamin-A) was well accepted with an average intake of 142 g/CU/day.

**Table 4:** Average intake (g/CU/day) of different food groups against recommended dietary intake (RDI) between Baseline (2014) and Endline (2017) in Koraput (N = 156).

Foods	Baseline (2014)	Endline (2017)	RDI
Cereals and millets	624.15	603.07	375
^**#**^Finger millet	70.37	80.01*	
^**#**^Maize	231.29	307.28	
Pulses and legumes	27.45	44.54*	75
^**#**^Pigeon Pea	-	58.85	
^**#**^Black gram	39.81	51.79	
^**#**^Green gram	49.23	57.72	
Leafy vegetables	57.5	124.79*	100
Other vegetables	115.86	245.2*	200
Roots and tubers	89.89	124.33*	200
^**#**^OFSP	-	141.88	
Fruits	86.59	124.33*	100
Fishes and sea foods	10.54	28.12*	
Meat and Poultry	15.56	11.52*	

^**#**^Sourced from home production through FSN crop intervention

*Significant @p<0.01.

In terms of frequency of consumption of different food groups, there was a marked increase in “daily” consumption of vegetables from 6 per cent households at baseline to 50 per cent at endline. Similarly, percentage of households consuming pulses and legumes, leafy vegetables, roots and tubers, fruits, fishes and milk, “twice or thrice a week” increased, suggesting increase in frequency of intake. These changes in food frequency pattern may be attributed both to increased availability as well as greater awareness following the awareness programmes on different aspects of nutrition.

#### Wardha

*Production diversity*. In Wardha, introduction of improved variety of pigeon pea resulted in seed yield that was twice as much as what the farmers used to get from local varieties (600 kg ha^-1^). None of the households in the survey sample (N = 158) were cultivating green gram or black gram at the baseline. However, at endline, 25 and 19 per cent of these households were cultivating the two crops with production of 505 and 658 kg ha^-1^, respectively (Table 5). Diversification in pulse production was also observed. Compared to baseline, percentage of households cultivating pigeon pea in endline was lower as farmers who wanted to take a *rabi* crop went in for short duration pulse crops (green gram and black gram), instead of a long duration crop of pigeon pea. The percentage of households cultivating sorghum and its production was higher as compared to baseline. In *rabi*, the micronutrient-dense improved varieties of wheat were grown by the same households at endline as in baseline but with twice the production that they used to get from local varieties. Similarly, cultivation of the wilt-resistant chickpea variety resulted in 37 per cent increased production. With regard to cultivation of fruits and vegetables, the proportion increased from 20 per cent households at baseline to 89 per cent at endline; households that grew only one or two vegetables had started growing different groups of vegetables. Around nine per cent of sample households were having poultry at endline with an average production of 50 eggs and 25 kg meat per household per year against nil during the baseline.

**Table 5:** Comparison of FSN crop interventions, cultivation and production status between Baseline (2014) and Endline (2017), Wardha (N = 158).

Crop interventions under FSN	Baseline	Endline
Sorghum		
% of households cultivating	6	26
^a^Production (kg/ha^-1^)	1000±131	2200±45
Pigeon pea		
% of households cultivating	65	41
Production (kg ha-1)	600±74	1268±22
Green gram		
% of households cultivating	0	25
Production (kg ha-1)	0	505±20
Black gram		
% of households cultivating	0	19
Production (kg ha-1)	0	658±15
Wheat		
% of households cultivating	16	16
Production (kg ha-1)	1500±157	3200±48
Bengal gram		
% of households cultivating	11	21
Production (kg ha-1)	658±63	900±12

^a^ indicates production figures in value ± standard deviation.

*Food consumption pattern*. The average consumption of cereals and pulses was found to have improved significantly after the intervention and met the RDI (cereals: 329g/CU/day at baseline to 382g/CU/day at endline against RDI of 375 g/CU/day; pulses: 52g/CU/day to 93g/CU/day against RDI of 75 g/CU/day). The consumption of other vegetables, green leafy vegetables and animal source foods particularly milk, also improved significantly, to the borderline of RDI. The percentage of households consuming more than 70 per cent of RDI of all food groups was seen to have increased at the endline, as seen in Table 6. This is also seen with regard to the crops promoted.

**Table 6:** Average intake (g/CU/day) of different food groups against recommended dietary intake (RDI) between Baseline (2014) and Endline (2017) in Wardha (N = 158).

Foods	Baseline (2014)	Endline (2017)	RDI
Cereals and millets	329.29	382.11*	375
^**#**^Wheat	234.87	272.54	
^**#**^Sorghum	150.00	245.52	
Pulses and legumes	51.69	92.88*	75
^**#**^Bengal gram	44.32	102.30*	
^**#**^Black gram	-	53.74	
^**#**^Green gram	-	82.97	
^**#**^Pigeon pea	38.67	117.72*	
Leafy vegetables	47.75	81.70*	100
Other vegetables	81.48	190.42*	200
Roots and tubers	33.52	55.95*	200
Fruits	11.31	91.20*	100
Fishes and sea foods	5.96	12.87*	
Meat and Poultry	21.4	28.68*	

^**#**^Source from home production through FSN crop intervention

*Significant @p<0.01.

Average intake of pigeon pea doubled, bengal gram intake increased 1.3 times, sorghum by 64 per cent and wheat by 16 per cent. Households were already consuming wheat twice a day as part of their regular diet and so, the change in intake was not much. It can also be seen that households had started consuming green gram and black gram from their own production as sole crop or intercrop. This increased quantity of home-produced foods suggests the feasibility of the FSN strategy promoted of growing food crops in commercial cropping patterns via inter cropping and by modifying the package and practices of food crops already being grown, for increased production.

In terms of pattern of frequency of food consumption, there was an increase in percentage of households consuming pulses and leafy vegetables and fruits ‘daily’; this might be due to increased availability of diversified legumes (pigeon pea, black gram, green gram and bengal gram) and variety of vegetables and fruits from backyard and community gardens. Introduction of backyard poultry also led to increase in the number of households consuming egg ‘daily’ from two at baseline to 17 at endline. Similarly, compared to baseline, an additional 26 percentage of households started consuming fish and sea foods, possibly an impact of better nutrition awareness leading to positive shift in frequency of their consumption.

#### Household dietary diversity

The number of food items consumed under different food groups as well as the percentage of households consuming more than 70 per cent of the RDI was better at endline in both the study sites. This is shown in Table 7. However, the increase was more in Wardha as compared to Koraput. In Wardha, the increase in the percentage of households consuming > 70% RDI was higher for all the three groups of vegetables and fruits and also for cereals and pulses and legumes by 20 and 55 per cent respectively.

**Table 7:** Comparison of no. of items consumed under each food group and percentage of households consuming ≥70% of RDI*.

Food groups	Koraput (N = 156)		Wardha (N = 158)	
	Baseline (2014)	Endline (2017)	Baseline (2014)	Endline (2017)
Cereals and millets				
No. of items	3	5	2	4
% of households#	94.8	96.8	20.6	40.6
Pulses and legumes				
No. of items	5	7	4	6
% of households#	0.6	16.9	12.9	67.1
Leafy vegetables				
No. of items	5	8	6	11
% of households#	9.1	33.1	4.5	22.6
Other vegetables				
No. of items	12	15	6	16
% of households#	12.3	39.0	0	41.9
Roots and tubers				
No. of items	5	5	2	5
% of households#	3.9	11.0	0	0^a^
Fruits				
No. of items	5	7	1	8
% of households#	24.7	36.4	0.6	33.5
Fishes and sea foods$				
No. of items	2	4	1	2
Meat and Poultry^$^				
No. of items	3	3	2	2

*Recommended Dietary Intakes (RDI) as per Dietary Guidelines for Indians, ICMR, 2011 [33].

#Households consuming ≥70% of RDI

$ % of households is not given as RDI is not available for these groups; a Though there was an increase in no. of HHs consuming roots and tubers at endline, none were consuming >70% of RDI, hence it is shown as ‘zero’.

Household dietary diversity score (HDDS) was calculated by number of food groups consumed per day. Table 8 gives the details. In Koraput, HDDS increased significantly at *p*<0.001 from 6.92 to 7.69 whereas in Wardha the change was very slight but significant. The percentage of households in Wardha having HDDS 9 increased from 5 per cent at baseline to 15 per cent at endline. In Koraput, households having HDDS 8 and 9 increased from 19 per cent and 5 per cent respectively at baseline to 29 per cent and 10 per cent respectively at endline, showing that the FSN interventions had improved the household dietary diversity.

**Table 8:** Percentage of households based on household dietary diversity score.

HDDS	Koraput (N = 150)		Wardha (N = 150)	
	Baseline (2014)	Endline (2017)	Baseline (2014)	Endline (2017)
Average HDDS	6.92	7.69**	7.53	7.74*
Dietary score	% of households			
5	4.1	2.0	0.6	0
6	26.7	17.2	8.1	6.7
7	43.8	39.5	30.4	30.7
8	19.2	29.4	54.7	46.0
9	5.5	10.1	5.0	15.3
10	0	1.4	0.6	1.3

Significant @

**p<0.001

*p<0.05.

#### Nutrition awareness

Endline focus group discussion revealed awareness among members of the community that balanced diet can improve health; and that the nutrients in food will address undernutrition, anemia and vitamin A deficiency. School children had better knowledge on sanitation, hygiene and importance of nutrients in foods [35,36]. Attitudinal and behaviour changes initiated by the CHFs were observed, with respect to consuming a balanced meal, spacing of meals, number of times a meal was consumed, sources of food that people accessed, setting up a nutri-garden to joining a community seed bank. An external evaluation of the nutrition awareness programmes conducted under the project observed ‘moderate’ impact in Koraput, with a large pool of CHFs joining the capacity building exercise with a genuine interest to learn about leveraging agriculture for nutrition and empowering themselves in the process. Compared to Koraput, a milder effect was observed in Wardha district, where understanding of the approach took time [37].

## Discussion

**Fig 2:**
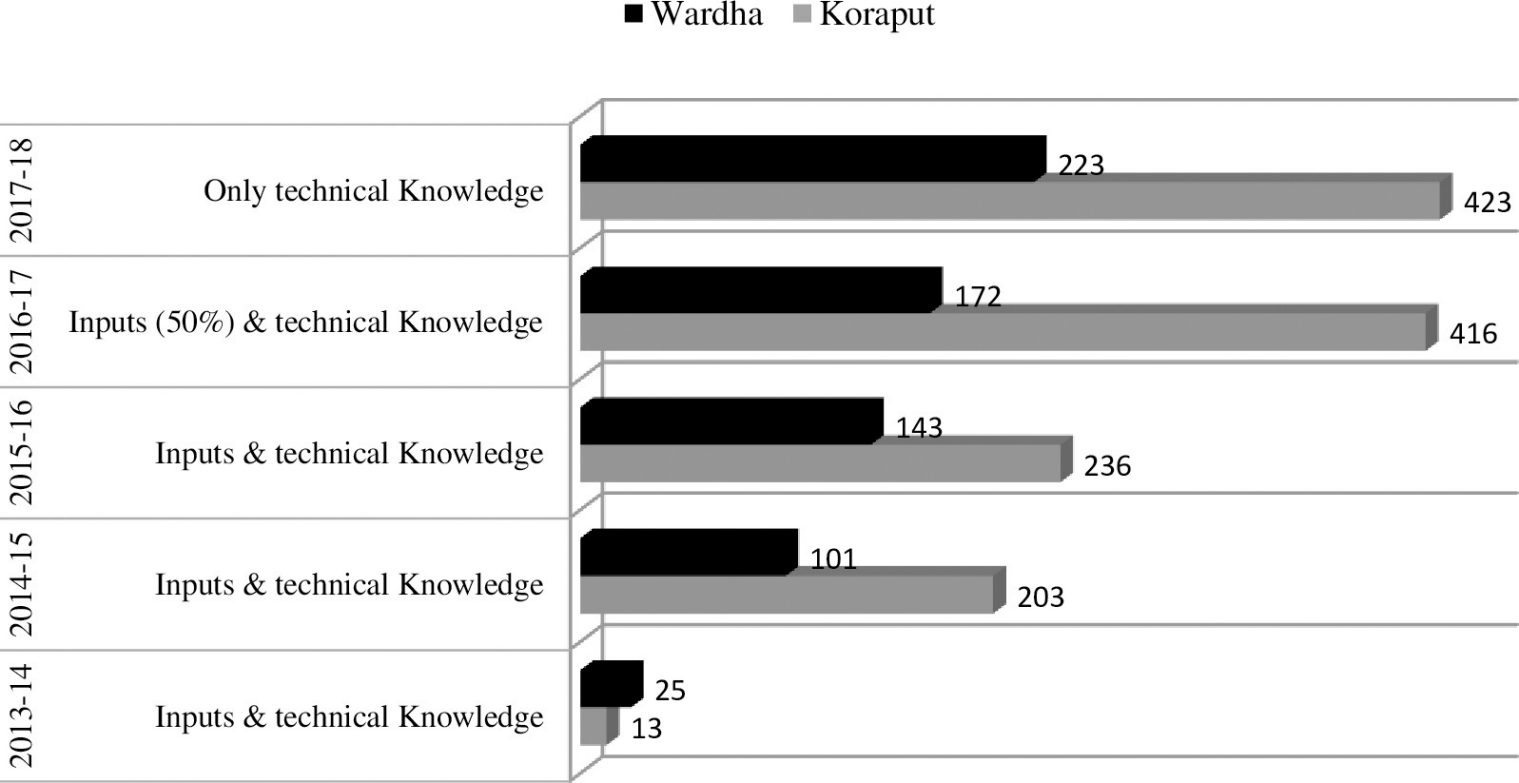
No. of farmers participating in FSN interventions (total households Koraput = 658, Wardha = 556).

Based on the identified disconnect between agriculture and human nutrition, this study shows potential pathways to overcome food and nutrition insecurity at household level. It demonstrated at both the study sites that an increase in production and greater diversity at the household level led to greater availability and increase in number and quantity of different food groups consumed and improved dietary diversity. The introduction of improved varieties and nutrient rich crops in the existing cropping systems along with nutrition gardens helped diversify the household food production basket to include nutrient-dense cereals, pulses, vegetables and fruits. Integration of poultry and fishery improved the access to animal food sources. The overall improvement in number of food items consumed and percentage of households consuming more than 70 per cent of RDI under each food group as well as household dietary diversity was better in both the study locations. This might be attributed to greater availability from encouraging households to cultivate food crops (pigeon pea, green gram, black gram and vegetables and fruits) in fields along with or in place of commercial crops (cotton, soybean) in Wardha whereas in Koraput, suitable package of practices for traditionally grown food crops (finger millet, green gram, black gram) and intercropping of pigeon pea as a *kharif* pulse crop in maize fields were established. At both study sites, increased food availability and diversity coupled with nutrition awareness helped the households improve food consumption pattern, move towards meeting the daily RDI, and ensure better nutrient intake [38]. This finding validates the relevance of the production to consumption pathway in both subsistence and commercial farming situations, suggesting greater availability of diverse foods from own production at farm level can lead to increased and more nutritious household food consumption. While the study findings are in contrast to studies like Kumar et al. [39] and Rosenberg et al. [40] that identified limited conversion of diverse agricultural production into overall dietary diversity, it is in line with several other studies that advocate potential of the production-led consumption approach. According to Herforth [41], crop diversity was significantly associated with household dietary diversity and also more closely related to household consumption from own-produced food than consumption of market-purchased food. More diverse production systems contributing to more diverse household diets for farming communities has been reported by Djokoto et al. [42]. A strong positive relationship between farm diversity and dietary diversity was also established by Jones et al. [43]. There is also published literature on specific interventions such as positive impact of home garden interventions on intermediate nutritional incomes such as dietary diversity and consumption of nutrient rich foods [44–47]; improved consumption of animal source foods through integration of fishery [48], and livestock [44,49] and combination of nutrition garden and poultry together with nutrition awareness, improving consumption of vegetables and eggs [50]. However, this study is perhaps one of the first in designing a system-wide farming intervention (including field crops, vegetables and fruits, and animal source foods) based on resource availability, accompanied by nutrition awareness, to enhance dietary diversity of households primarily involved in agriculture and allied activities. Agriculture being the major source of livelihood in the study areas facilitated the FSN approach. It not only improved the crop yield but also mainstreamed the nutrition dimension in the choice of field crops, fruits and vegetables. The importance of on-farm and natural/wild food environments as part of food environment for producers and rural residents has also been reported by several other studies [51–53].Starting with a small number of farm households and providing both input and technical knowledge support for on-farm demonstrations in 2013–14, farmers were facilitated with only technical knowledge (technical knowledge here indicates advisory on crop management practices given to farmers as and when required; there was no cash or any input support such as seeds or fertilizers) in 2017–18 and there is evidence of uptake within the core study villages, depicted in Fig 2. The linkage between agriculture and nutrition and objective of the FSN study had been explained to them at village meetings, training programs, technical sessions, plant health clinics, animal health camps, programmes on value addition, focus group discussions, on-farm demonstrations, farmer’s field day and through exposure visits.

The uptake of FSN interventions had also expanded beyond the core group of villages by 2017, to 25 more villages in Wardha and 18 more villages in Koraput, reaching out to more households. Farm men and women emerged as spokespersons of the FSN approach within the community and at different stakeholder forums. The expedited uptake of FSN interventions in the study areas is attributable to farmers’ realization of economic benefits via, a) retaining the produce for self-consumption (thereby reducing expense on food purchase) and selling the surplus if available. Further, comparison of food consumption pattern and dietary diversity between the same set of households at the baseline and endline of the study reveals the impact of the FSN interventions. The CHF approach to promote nutrition awareness created a learning space for the community and built a community resource network for integrating learner-centred nutrition literacy in the agriculture-dependent community. Evidence of this has been demonstrated in Narayanan and Rao [54]. Considering the sociocultural sensitivity of the study population, this study was conducted as a feasibility study without any control group. In terms of other externalities, there were no major government initiatives with regard to agriculture in both the study areas during the period of study. The gradual changes seen over the period 2014 to 2017, in terms of production diversity and dietary diversity through promotion of FSN interventions including nutrition awareness, indicates the feasibility and acceptability of the FSN approach. While the evidence generated through this study provides a framework for designing nutrition-sensitive farming systems, the feasibility has to be demonstrated in different agro-ecological zones of the country and scalability and sustainability established. Further study is also required to understand the gendered impact of the interventions. Qualitative research in both study locations during the course of the FSN study for instance indicated that women’s time for care work is reduced during the peak agriculture seasons. There is also differential impact between different social groups [55]. At the same time, the improved agriculture practice of line transplanting that was introduced reduces the need for frequent weeding, an activity generally performed by women.

The M S Swaminathan Research Foundation has been engaging in advocacy with agriculture universities and policy makers in different states, to promote the approach [56]. Soil health is an important aspect that was not directly addressed under the current study and must be included in future research programs under FSN. While the millet and pulse crops promoted under FSN are rainfed crops and generally regarded as climate resilient, their contribution towards mitigating impact of climate change needs further study. Post-harvest processing and value addition with minimum loss of nutrients are also aspects that have to be addressed, to ensure shelf life and longer period of availability. This will give an impetus to the development of local value chains. The current study essentially focused on crop-based interventions; however, based on the resource base available, agro-forestry, dairy or fishery, for instance can be an important component of the FSN design. Interlinking the different components under an integrated farming approach will provide further impetus.

## Conclusions

Farming System for Nutrition is a location-specific, inclusive approach based on resource endowments and a specific environment, to address the nutritional needs of households. It is a flexible approach that takes into account the nature of resource endowments available, specificities in environment and nutrition problems, because of which farmers can decide on possible combinations of different components of FSN. The approach can be seen as a subset of the larger canvas of agroecology and sustainable food systems, with explicit focus on addressing household nutrition security. The principles of having a nutrition focus, context assessment, nutrition awareness and design to address nutrition deficiencies, however, will be critical in all cases. Although some nutrient gaps will need to be met by other means such as food fortification, agricultural policy should have inbuilt capability to improve nutrient adequacy through greater productivity of available crops, crop diversification or animal husbandry, depending on local food consumption practices. Generating evidence of impact and building policy support for promotion of available bio-fortified crop varieties (e.g. zinc fortified rice or iron fortified sorghum) is also an urgent requirement in this context. In addition, agricultural policy can make a real impact by considering appropriate means to incentivize additional production and consumption of nutrient-dense foods like millets and pulses, particularly if the crop is not currently produced or consumed in large amounts. This will have to be accompanied by nutrition awareness strategies and campaigns to create consciousness and generate consumer demand. The potential of the approach to influence and improve intermediate outcomes such as dietary diversity and the consumption of nutrient-rich foods irrespective of agro-ecological differences as demonstrated by the study, highlight need for greater support and research in this important area. The results of the study not only add to the evidence for nutrition-sensitive agriculture approach discussed in Ruel et al. (2018) [21], but also contribute to the larger discussion around agro-ecology and food system discussed by Kerr et al. 2019 [57]. The recent experience of the COVID-19 pandemic and breakdown of supply chains following lockdown in India, further reinforces the need for decentralized approaches and local value chains to strengthen community resilience. Practice of the FSN approach by smallholder farmers who constitute a large majority of farmers, can be an important means to move towards Sustainable Development Goal 2 of Zero Hunger.

## Supporting information

S1 Table Details on crop based demonstrations at study sites. (PDF)

S1 File Focus group discussion checklist. (PDF)

S2 File Survey questionnaires. (PDF)
