# Supplementary material for: Farming System for Nutrition-a pathway to dietary diversity: Evidence from India
Source: PLoS One. 2021 Mar 18;16(3):e0248698. doi: 10.1371/journal.pone.0248698 (PMC7971902; doi:10.1371/journal.pone.0248698)
Supplement: S1 Table — (PDF) [file pone.0248698.s001.pdf]

S 1 Table. Details on crop based demonstrations at study sites (2013-16)

Koraput

Year	Season	On farm demonstrations	No. of farmers	Conclusion
2013-14	Kharif	Improving fertilizer-use efficiency through Urea Super Granules (USG) in rice	3	Rice crop under USG had higher yield contributing parameters and recorded (14-28) % higher grain yields than that under prilled urea, irrespective of varieties.
		Demonstrations on mixed cropping	4	Crop combinations with finger millet and black gram were performing better compared to combinations with sorghum and rice.
		Demonstration on orange flesh sweet potato (OFSP) cultivation	1	The tuber yield was 5.3 t ha^{-1} as well as served as a demonstration plot to other farmers.
	Rabi	Yield enhancement of rice with and without Urea Super Granule	1	Yield affected due to low temperature.
2014-15	Kharif	Fertilizer Deep Placement in Rice with USG	15	Rice varieties under USG had higher yield (9-18)% as compared to prilled urea.
		Improved production practices of pulse-finger millet cropping system	10	Intercropping black gram with finger millet in a ratio 2:6 showed superior yield performance, as compared to mixed broadcasting of black gram and finger millet.
	Rabi	Improved production practices of green gram	5	Improved variety (SML-668) gave 14% higher seed yield than farmers' varieties (425 kg ha^{-1}).
		Improved production practices of black gram	35	Improved variety TK94-2 (351 kg ha^{-1}) gave 17% higher seed yield than existing variety NVL-7 (300 kg ha^{-1}).
2015-16	Kharif	Improved production practices of finger millet	10	Under improved agronomic practices, GPU-67 produced 13% higher yield than that of farmer's varieties (1832 kg ha^{-1}).
		Improved production practices of pulse-finger millet intercropping	10	It was observed that black gram + finger millet and pigeon pea + finger millet in 2:6 ratio and under recommended agronomic practices had higher crop yield in comparison to mixed broadcasting.
		Pulse-maize intercropping with improved agronomic practices	13	Pigeon pea tender and matured pods were additional yield with maize from the same field during same time.
		Introduction of shorter duration rice varieties	31	Short duration varieties showed superior yield performance, as compared to most of the existing popular varieties in the area.
		Cultivation of orange flesh sweet potato in fields	22	Households kept the OFSP produce for consumption.
	Rabi	Demonstration on improving the production and productivity of finger millet	20	The grain yield of Bhairabi was only 8 per cent higher than farmer's variety (1414 kg ha^{-1}).
		Enhancing pulse production through intercropping of Dolichos bean and French bean with maize	19	Yield loss was there due to adverse weather condition.
		Improved production practices of green gram	48	SML-668 (495 kg ha-1) gave 17 per cent superior yield than NVL-585 (412 kg ha-1) and 7 per cent superior yield than Nayagarh local (458 kg ha-1).
		Improved production practices of black gram	16	TK94-2 (368 kg ha-1) gave 15 per cent superior yield than NUL-7 (312 kg ha-1).

Wardha

Year	Season	On farm demonstrations	No. of farmers	Conclusion
2013-14	Kharif	Evaluating the performance of desi cotton variety (Suraj)	20	Cotton lint yield for Suraj variety was 37 per cent less compared to Bt cotton hybrids (1370 kg ha^{-1}).
		Assessing the performance of improved variety of pigeon pea (Durga) as inter crop in cotton (2: 6)	15	Improved pigeon pea variety 'Durga' had 26% higher seed yield than local pigeon pea varieties grown by farmers under intercropping with cotton (263 kg ha^{-1}).
		Assessing the performance of improved variety of soybean (N-15)	10	Soybean yield was affected due to unseasonal rainfall.
	Rabi	Assessing performance of zinc and iron rich wheat varieties	6	The average grain yield of zinc and iron rich wheat varieties were 3239 and 3594 kg ha^{-1}respectively; while the grain yield of local varieties were 1375 kg ha^{-1}.
		Evaluating the performance of improved varieties of chick pea (Jacki-9218 and PKV-Kabuli)	4	The grain yields of improved varieties were 25-30% higher than that of local farmer varieties.
		Assessing the performance of improved linseed varieties (NL-260)	2	The improved variety recorded 53 per cent higher yield than farmers' varieties.
		Assessing the performance of improved variety of mustard (Shatabdi)	5	The seed yield of 660 kg ha^{-1} was recorded.
2014-15	Kharif	Evaluating the performance of desi cotton variety (Suraj)	30	The average lint yield was 1400 kg ha^{-1}.
		Assessing the performance of improved variety of pigeon pea (PKV-Tara) as sole crop and inter crop in cotton (2: 6)	30	Average seed yield was reported to be 700 and 425 kg ha^{-1} from sole and inter cropping, respectively.
		Assessing the performance of improved variety of Soybean (N-15)	11	The average seed yield was reported to be 1774 kg ha^{-1}.
		Assessing the performance of improved variety of sorghum	17	Average seed yield was reported to be 994 kg ha^{-1}.
		Assessing the performance of improved variety of green gram as an intercrop with cotton (2: 6)	29	An average seed yield of 420 kg ha^{-1} was reported.
	Rabi	Assessing performance of zinc and iron rich wheat varieties	27	The average grain yield was 71 per cent higher than that of local varieties.
		Assessing the performance of improved variety of chick pea (Jacki-9218)	15	Jacki-9218 had 16 per cent higher grain yield than farmers' varieties.
2015-16	Kharif	Evaluating the performance of three desi cotton varieties (Suraj, NH-615, Phule Dhanwantary)	35	The performance of desi varieties ranked in the order of NH-615 > Suraj > Phule Dhanwantary.
		Improved production practices of pigeon pea variety NTL-900	22	The improved variety had seed yield of 1533 kg ha^{-1}.
		Assessing the performance of improved variety of pigeon pea and green gram as intercrop with cotton	25	Pigeon pea variety NTL-900 and green gram variety iskopargaon had an average seed yield of 1313 and 554 kg ha^{-1}, respectively.
		Improved production practices of sorghum	41	The average yield of CSV-20 was reported to be 320 kg ha^{-1}, 33 per cent lower as compared to most of the popular farmers' hybrids.
	Rabi	Improved production practices of chickpea	20	The yield of Jacki – 9218 was found to be 15% higher than the farmers' varieties (782 kg ha^{-1}).
		Improved production practices of onion (improved variety and package of practices)	19	The onion yield of improved variety (Bhima Super) was 94 per cent higher than farmer's variety.
		Performance of nutrient dense wheat varieties	35	The nutrient dense wheat varieties were 15-20 per cent higher than farmer's varieties.
