# Supplementary material for: Farming System for Nutrition-a pathway to dietary diversity: Evidence from India
Source: PLoS One. 2021 Mar 18;16(3):e0248698. doi: 10.1371/journal.pone.0248698 (PMC7971902; doi:10.1371/journal.pone.0248698)
Supplement: S1 File — (PDF) [file pone.0248698.s002.pdf]

S 1 File. Focus Group Discussion Checklist

Leveraging Agriculture for Nutrition in South Asia (LANSA)

Farming Systems for Nutrition (FSN) Study

Focus Group Discussion

2017

M S Swaminathan Research Foundation, Chennai, India

Focus Group Discussion - Checklist

1. Were people familiar with the fact that nutrition and good health is linked to the food we eat?
2. Which interventions are most popular?
3. Due to the interventions, what happened
    a. Are they, specifically women, working longer hours?
    b. Are more members from the household working now due to the intervention?
    c. Has something else changed, such as use of crop residue for fuel or feed?
    d. Are there more biofortified crops? For instance, drumstick (prompt only if no response)
    e. Has any intervention also led to an increase in soil nutrients?
    f. Is there an increase in home grown consumption?
    g. Are they consuming more pulses than before?
    h. How are pulses consumed?
    i. Are they consumed all through the year?
    j. What about processing?
    k. Are you also more aware about storage?
4. What are the challenges?
5. Nutrition gardens
    a. Do nutrition gardens help in increasing home consumption of fruits and vegetables?
    b. Do you get enough throughout the year?
    c. Have you told others?
    d. Do you get surplus? If yes, what do you do with surplus from home/community garden?
    e. How do you get seeds?
    f. What are the challenges?
6. What percentage of expenditure of the total expenditure is on food?
7. Has this come down after interventions?
8. Are producers aware of the access to markets? Where do they sell - local market or somewhere further? For instance, if in some villages, it is known that there is a weekly market, is that incentive to produce?

9. Are they aware of Minimum Support Prices (MSP)?
10. Do the producers prefer to sell in the market and source it from the market itself, rather than home grown i.e. is the income effect more pronounced?
11. Are you or anyone else you know growing nutritious food with your own crops?
12. Are there any strengths of the programme?
13. Are there any weaknesses of the programme? Do you have any suggestions on how to improve this programme?
14. Have you been telling your friends and family about the interventions?
15. How will you get seeds in the future?
16. Will you continue growing nutri-foods even after the project is over?
17. Have there been any changes in the type of sanitation and use of cooking fuel in the past 3 years? If yes, get details.

Nutrition Awareness

1. Have you attended the nutrition awareness meetings/programmes conducted by MSSRF?
2. Was it informative? Are you able to follow it in your day today life?
3. Do you know the benefits of balanced diet and food items? Were you able to bring changes in your daily consumption pattern? If yes, list some.
4. Effects of anemia in women and VAD in children. What are the foods that are good for anemia and VAD?
5. Have you seen the nutrition calendar with pictures drawn by school children? Was it useful?
6. Did your child share any messages related to health, nutrition and diet and WASH?
7. Are you aware of community hunger fighters/community resource persons? Have you heard that they have undergone trainings on nutrition and linking agriculture to nutrition?
8. What are the messages that you have heard from them?
9. Have you discussed anything related to food and nutrition with CHF?
10. Do you think that creating a CHF at community level is useful to make change in nutrition and health of population?
