# Supplementary material for: Farming System for Nutrition-a pathway to dietary diversity: Evidence from India
Source: PLoS One. 2021 Mar 18;16(3):e0248698. doi: 10.1371/journal.pone.0248698 (PMC7971902; doi:10.1371/journal.pone.0248698)
Supplement: S2 File — (PDF) [file pone.0248698.s003.pdf]

S 2 File. Survey Questionnaires

Leveraging Agriculture for Nutrition in South Asia (LANSA)

Farming Systems for Nutrition (FSN) Study

Endline Survey

2017

M S Swaminathan Research Foundation, Chennai, India

Consent Statement

This survey is part of a study conducted by M S Swaminathan Research Foundation under the project Leveraging Agriculture for Nutrition in South Asia (LANSA). You are requested to participate in this voluntary interview. The information provided by you will be used only for the purpose of our research.

Respondent Name

Signature

Schedule 1: Household Characteristics

Dist Name………………………. Village Name…………………..…. HH ID________Date of Interview____ /____ /____

1.1 Name of the head of the household………………………………………………………. Respondent Name………………………………………. Investigator Name…………………………………

1.1a Caste…………………………… 1.1b Sub-caste …………………………………. 1.1c Household Size …………….. (Total) Male………….. Female………..

1.2 Particulars of the household members

S.No	Particulars	101	102	103	104	105	106	107	108	109	110
1	**Name**										
2	**Gender (M=1; F=2)**										
3	**Age***	____ . ____	____ . ____	____ . ____	____ . ____	____ . ____	____ . ____	____ . ____	____ . ____	____ . ____	____ . ____
4	**D.O.B(dd/mm/yyyy)**										
5	**Relationship to Head Code**										
6	**Education Code**										
7	**Physiological Status code**										
8	**Major Occupation Code**										
9	**Subsidiary occupation**										
10	**Physical ActivityCode**										
11	**Consumption Units**										
12	**Whether migrated in the last year*(Yes=1;No=2)***										
13	**Whether remittance sent (*Yes=1;No=2)***										
14	**If yes, how much? (In Rs)**										
15	**Whether participated in MGNREGS*(Yes=1;No=2)***										
16	**Number of days**										
17	**Wages earned MGNREGS**										
18	**Remarks**										

*Age in completed years; Up to the nearest month in case of children below 5 years of age

**Code for Caste : ** SC=1; ST=2; OBC=3; Others=4

Code forS.No(5) – Relationship to the Head of the Household

Head=1; Spouse=2; Children=3; Son/daughter-in-law=4; Grandchildren=5; Parents=6; Siblings=7; Parents-in-law=8; Niece/Nephew=9; Grandparents=10; Any Other=11

Code for S.No(6) - Education Code

Illiterate	=1	Completed Secondary (10thStd)	=6	Not Applicable (<4 years)	= 11
Pre-School/ICDS (4-6years)	=2	Completed Higher Secondary (12thStd/+2)	=7		
Less than primary	=3	Graduate & above	=8		
Completed Primary (5thStd)	=4	Vocational/technical courses	=9		
Completed Middle School (8thStd)	=5	Not going to school (6-14yrs)	=10		

Code for S.No (7) – Physiological Status

Women in the age group 15-45yrs : NPNL (Non-pregnant Non-lactating)=1; Pregnant=2; Lactating (up to 12 months)=3
Children <2 yrs of age : Only breast fed=4; Breast fed+water=5; Breast fed+Complementary feed=6; Not breast fed=7
All Others* : Not Applicable=9

Code for S.No(8) & (9)

Unemployed=1; Not in labour force (Student, Housewife, Elderly persons)=2; Cultivation=3;
Allied activities (livestock, fisheries & forestry)=4; Agri wage labourer=5; Non-agri labourer=6;
Manufacturing & processing (Agri)=7;Manufacturing & processing (Non-agri)=8; Trade & transport (Agri)=9; Trade & transport (Non-Agri)=10; Services=11; Any Other=12
[Tea shop/grocery shop/vegetable seller comes under code-10, Disari/THP/TBA comes under code-12]

Code for S.No (10) – Physical Activity

Sedentary –Student, Landlord, Service, Business, Housewife, Postman, Teacher etc =1
Moderate – Ag. labour, Other labour, Cultivator, Artisans, masons, Servant maid, Tailor, Rickshaw puller etc = 2
Heavy – Blacksmith, Stone cutter, Wood cutter, Mine worker etc = 3

Code for Col (11) – Consumption Units

Adult male (≥ 18yrs) – Sedentary=1.0 Moderate=1.2 Heavy=1.6
Adult female (≥ 18yrs)- NPNL : Sedentary=0.8 Moderate=0.9 Heavy=1.3
Pregnant : Sedentary=0.9 Moderate=1.0 Heavy=1.4
Lactating : Sedentary=1.0 Moderate=1.1 Heavy=1.5
Adolescents & Children (<18yrs):
16 -17 yrs : Boys=1.2, Girls=0.9 7 - 9 yrs (B+G)=0.9
13 -15 yrs : Boys=1.1, Girls=1.0 4 - 6 yrs (B+G)= 0.7
10 -12 yrs : Boys=1.0, Girls=0.9 1 - 3yrs (B+G)= 0.5 < 1 year (B+G)=0.0

1.3 House Type ☐

1. Kuccha 2. Semi-Pucca 3. Pucca

1.4 Source of Drinking water ☐

1. Dug well2. Piped Water3. Tube well/bore well4. Any other _______

1.5 What kind of toilet facility does this household use? ☐

Open defecation=1; Closed toilet=2; Any Other (Specify………………)=3

1.6 What type of fuel does this household mainly use for cooking?

Firewood=1; Crop residue=2; Kerosene=3; Cow dung=4; LPG=5; Bio-gas=6;

Any Other (Specify…………………………………..)=7

Primary Source ☐ Secondary Source ☐

1.7 Do you have Electricity Connection? ☐

1. Yes 2. No

1.8 Do you have crop insurance/ weather insurance etc? Y=1; N=2 ☐

If Yes, fill in the table

S.No	Name	Individual ID	Crop insurance=1; Flood insurance=2; Drought insurance=3	Remarks
(1)	(2)	(3)	(4)	(5)
1				
2				

1.9 Household Expenditure (last 12 months)

S.No	Items	Value (Rs.)
1	Education(Books, stationery, fees etc)	
2	Medical expenses	
3	Any other expenditure (Clothes/Marriage/ Death/ Family function)	
4	Total expenditure food & non food (Last Month)	

1.10Asset Details/ Asset Purchase Details

S.No	Asset	Do you own any? (As on date) Y=1; N=2	Number as on date	Purchased any in the last one year? Y=1; N=2	If Yes, Value (Rs.)	S.No	Asset	Do you own any? (As on date) Y=1; N=2	Number as on date	Purchased any in the last one year? Y=1; N=2	If Yes, Value (Rs.)	S.No	Asset	Do you own any? (As on date) Y=1; N=2	Number as on date	Purchased any in the last one year? Y=1; N=2	If Yes, Value (Rs.)
(1)	(2)	(3)	(4)	(5)	(6)	(7)	(8)	(9)	(10)	(11)	(12)	(13)	(14)	(15)	(16)	(17)	(18)
	**Household**						**Agriculture**						**Animal Husbandry**				
1	House					19	Ag. Land (Acre)					37	Milch Cattle				
2	Non-ag. Land(Area)					20	Tube wells					38	Cattle				
3	TV					21	Open wells					39	Milch Buffalo				
4	Air Cooler					22	Tanks and ponds					40	Buffalo				
5	Radio					23	Electricity Pumps					41	Goat				
6	Electric Fan					24	Diesel Pumps					42	Pig				
7	Refrigerator					25	Drip irrigation					43	Poultry				
8	Cooking appliances					26	Sprinklers					44	Duck				
9	Mattress/Bed					27	Sprayer					45	Bullocks				
10	Furniture					28	Tractor					46	Sheep				
11	Bicycle					29	Tractor trolley					47	Animal shed				
12	Motor Cycle					30	Bullock Cart					48					
13	Mobile					31	Thresher					49					
14	Jewellery					32	Seed driller					50					
15	LPG Cylinder					33	Power Tiller					51					
16	Hand pump					34	Generator					52					
17						35	Storage facility*					53					
18						36						54					

*Storage facility means shed/ godown etc. outside the house. Does not include bin or bags stored within the house

1.11 Land details (in Acres)

Land Type	Total Own land	Irrigated					Unirrigated/ Rainfed				
		Own land	Leased in	Leased out	Fallow land	Encroached land	Own land	Leased in	Leased out	Fallow land	Encroached land
(1)	(2)	(3)	(4)	(5)	(6)	(7)	(8)	(9)	(10)	(11)	(12)
Light											
Medium											
Heavy											
Total											

Note 1: Own land is inclusive of leased out land and fallow land
Note 2: Own land should not include leased in land and Encroached land

Schedule 2: Agriculture, Animal Husbandry & Home Garden Details

Village Name…………………….…. HH ID________Date of Interview____ /____ /____Respondent Name………………………………. Investigator Name………………………………….

2.1.1 Household Farm Details in *Kharif* (For the last 12 months) Yes=1; [ ] No=2

2.1.1a Total Operational Land ……………………. (Acres) (***Note: Operational land includes own land as well as leased in – irrigated and rainfed***)

S.No	Crop Name	Crop ID	Area (Acres)			Land type	Border/ Field (only for veg)	Total Expenses (in Rs)	Total Output (Qtl.kg)	Mkt Value/ MSP (Rs.)	Qty - Self Cons (Qtl.kg)	Qty – Sold (Qtl.kg)	Price/ Qtl (Rs.)	Qty – Seed (kgs)	Value of seeds/Unit	Value of By Product (Rs.)
			Irrigated	Rainfed	Total											
(1)	(2)	(3)	(4)	(5)	(6)	(7)	(8)	(9)	(10)	(11)	(12)	(13)	(14)	(15)	(16)	(17)
1																
2																
3																
4																
5																
6																
7																
8																
9																
10																
11																

Codes for (7) Land type *1 light 2 medium 3 heavy* **Codes for (8)***Only for vegetables grown in 1 field 2 Border of field 3 In lines(with in field) 4 Any other (specify)____*

2.1.2 Household Farm Details in *Rabi* (For the last 12 months) Yes=1; ☐ No=2

2.1.2a Total Operational Land ……………………. (Acres) (***Note: Operational land includes own land as well as leased in – irrigated and rainfed***)

S.No	Crop Name	Crop ID	Area (Acres)			Land type	Border/ Field (only for veg)	Total Expenses (in Rs)	Total Output (Qtl.kg)	Mkt Value/ MSP (Rs.)	Qty - Self Cons (Qtl.kg)	Qty – Sold (Qtl.kg)	Price/ Qtl (Rs.)	Qty – Seed (kgs)	Value of seeds/Unit	Value of By Product (Rs.)
			Irrigated	Rainfed	Total											
(1)	(2)	(3)	(4)	(5)	(6)	(7)	(8)	(9)	(10)	(11)	(12)	(13)	(14)	(15)	(16)	(17)
1																
2																
3																
4																
5																
6																
7																
8																
9																
10																
11																
12																
13																

Codes for (7) Land type *1 light 2 medium 3 heavy* **Codes for (8)***Only for vegetables grown in 1 field 2 Border of field 3 In lines(with in field) 4 Any other (specify)____*

2.2 Livestock Details (For the last 12 months)Yes=1; No=2 ☐

S.No	Items		Cow	Buffalo	Goat	Sheep	Piggery	Poultry	Duckery	Ox/ Bullocks	Fisheries
(1)	(2)		(3)	(4)	(5)	(6)	(7)	(8)	(9)	(10)	(11)
1	Number (as on date)										
2	How many milch animals										
3	How many Used for agriculture/labour purpose										
	Expenses										
4	Purchase of animals (Rs.)										
5	Labour (Rs.)										
6	Feed (Rs.)										
7	Infrastructure (eg. Shed) (Rs.)										
8	Veterinary charges (Rs.)										
9	Interest on loan (Rs.)										
10	Other expenses (Rs.)										
11	Total (Rs.) (4 to 10)										
	Receipts										
12	Milk	Total Output (Ltrs)									
13		Self-cons - Qty (Ltrs)									
14		Distribution as gift (Ltrs)									
15		Sale - Qty (Ltrs)									
16		Value (Rs./Ltrs)									
17		Total Value (12*16)									
18	Egg	Total Output (Nos.)									
19		Self-cons - Qty (Nos.)									
20		Distribution as gift (Nos.)									
21		Sale - Qty (Nos.)									
22		Value (Rs./Nos.)									
23		Total Value (18*22)									
24	Meat	Total Output (Kg)									
25		Self-cons - Qty (Kg)									
26		Distribution as gift (Kg)									
27		Sale - Qty (Kg)									
28		Value (Rs./Kg)									
29		Total Value (24*28)									
30	Fish	Total Output (Kg)									
31		Distribution as gift (Kg)									
32		Self-cons - Qty (Kg)									
33		Sale - Qty (Kg)									
34		Value (Rs./Kg)									
35		Total Value (30*34)									
36	By-products (Rs.)										
37	Sale of animal (Rs.)										
38	Other receipts (Rs.)										

2.3.1 Home Garden Details (For the last 12 months)Yes=1; No=2 ☐

2.3.1a Total Home Garden Area in *Kharif* (square feet) _________

			Expenses (Rs.)									*Receipts*				
**S.No**	**Item**	**Code**	**Seeds/ Seedling/ plt.matl.**	**Manure**	**Fertiliser**	**Pesticides/ Insecticides (Organic)**	**Pesticides/ Insecticides (Inorganic)**	**Irrigation**	**Infrastructure (eg. Fence)**	**Other expenses**	**Total expenses**	**Output (Kg)**	**Self-cons (Kg)**	**Distribution as gift (Kg)**	**Qty - Sold (Kg)**	**Price/Kg (Rs.)**
(1)	(2)	(3)	(4)	(5a)	(5b)	(6a)	(6b)	(7)	(8)	(9)	(10)	(11)	(12)	(13)	(14)	(15)
1																
2																
3																
4																
5																
6																
7																
8																
9																
10																
11																
12																
13																
14																
15																
16																
17																
18																
19																
20	**Total Expenses (Rs.)**															

Column 4 - MSSRF Kit (Rs.10)

2.3.2 Total Home Garden Area in *Rabi* (square feet) _________

S.No	Item	Code	*Expenses (Rs.)*									*Receipts*				
			Seeds/ Seedling/ plt.matl.	Manure	Fertiliser	Pesticides/ Insecticides (Organic)	Pesticides/ Insecticides (Inorganic)	Irrigation	Infrastructure (eg. Fence)	Other expenses	Total expenses	Output (Kg)	Self-cons (Kg)	Distribution as gift (Kg)	Qty - Sold (Kg)	Price/Kg (Rs.)
(1)	(2)	(3)	(4)	(5a)	(5b)	(6a)	(6b)	(7)	(8)	(9)	(10)	(11)	(12)	(13)	(14)	(15)
1																
2																
3																
4																
5																
6																
7																
8																
9																
10																
11																
12																
13																
14																
15																
16																
17																
18																
19																
20	**Total Expenses (Rs.)**															

Column 4 - MSSRF Kit (Rs.10)

Schedule 3: Household Food Consumption Pattern

Dist Name………………………. Village Name…………………..…. HH ID __ __ __ __ Respondent Name………………………………………………. Date of Interview__ __ /__ __ /__ __

No. of Adult Members…………… No. of Children …………… Total CU __ __ . __ (Please enter this from Schedule 1)

S.No	Food Group	Food Code	Frequency of consumption (Code)	Raw amounts consumed (g)		Purchased from PDS	Home grown		Purchased from market		Collected from other sources		Market Price (Rs./Kg)	Remarks
				Per HH/Day	Per CU/Day	Qty (Kg/Ltr)	Qty (Kg/Ltr)	Ref Per	Qty (Kg/Ltr)	Ref Per	Qty* (Kg/Ltr)	Other source code		
(1)	(2)	(3)	(4)	(5)	(6)	(7)	(8)	(9)	(10)	(11)	(12)	(13)	(14)	(15)
	**Cereals and Millets**	1001												
1	Rice (raw milled)	12												
2	Wheat	21												
3	Rice Flakes	14												
4	Semolina (Suji)	24												
5	Puffed rice	15												
6	Ragi/finger millet	8												
7	Sorghum	4												
8	Foxtail Millet	7												
9	Little millet	16												
10	Maize (Tender)	6												
11	Maize (Dry)	5												
12														
13														
	**Pulses and Legumes**	1002												
14	Bengal gram Whole	28												
15	Bengal gram dhal	29												

Code for Col (4) – Frequency of Consumption
Daily=1; Twice /thrice a week=2; Once a week=3; Once in fifteen days=4; Once in a month=5; Occasionally=6
Code for Col (9), Col (11)
Daily=1; Twice /thrice a week=2; Once a week=3; Once in fifteen days=4; Once in a month=5; Occasionally=6

Code for Col(13)
Forest=1; Relatives/friends=2; Agriculture land=3; ICDS=4; Kind =5; Any other=6
* Total quantity collected during the last quarter

S.No	Food Group	Food Code	Frequency of consumption (Code)	Raw amounts consumed (g)		Purchased from PDS	Home grown		Purchased from market		Collected from other sources		Market Price (Rs./Kg)	Remarks
				Per HH/Day	Per CU/Day	Qty (Kg/Ltr)	Qty (Kg/Ltr)	Ref Per	Qty (Kg/Ltr)	Ref Per	Qty* (Kg/Ltr)	Other source code		
(1)	(2)	(3)	(4)	(5)	(6)	(7)	(8)	(9)	(10)	(11)	(12)	(13)	(14)	(15)
16	Bengal gram roasted	30												
17	Blackgram dhal	31												
18	Greengram whole	34												
19	Greengram dhal	35												
20	Pigeon pea/red gram dhal	44												
21	Peas green	40												
22	Peas dry	41												
23	Rajmah	43												
24	Lentil	38												
25	Rice bean dhal	465												
26	Horse gram	36												
27	Cow pea	32												
28	Broad Bean	33												
29														
30														
	**Leafy Vegetables**	1003												
31	Curry leaves	77												
32	Coriander leaves	75												
33	Amaranthus	50												
34	Indian Spinach	93												
35	Radish leaves	103												
36	Drumstick leaves	78												

Code for Col (4) – Frequency of Consumption
Daily=1; Twice /thrice a week=2; Once a week=3; Once in fifteen days=4; Once in a month=5; Occasionally=6
Code for Col (9), Col (11)
Daily=1; Twice /thrice a week=2; Once a week=3; Once in fifteen days=4; Once in a month=5; Occasionally=6

Code for Col(13)
Forest=1; Relatives/friends=2; Agriculture land=3; ICDS=4; Kind =5; Any other=6
* Total quantity collected during the last quarter

S.No	Food Group	Food Code	Frequency of consumption (Code)	Raw amounts consumed (g)		Purchased from PDS	Home grown		Purchased from market		Collected from other sources		Market Price (Rs./Kg)	Remarks
				Per HH/Day	Per CU/Day	Qty (Kg/Ltr)	Qty (Kg/Ltr)	Ref Per	Qty (Kg/Ltr)	Ref Per	Qty* (Kg/Ltr)	Other source code		
(1)	(2)	(3)	(4)	(5)	(6)	(7)	(8)	(9)	(10)	(11)	(12)	(13)	(14)	(15)
37	Cabbage	66												
38	Spinach	110												
39	Pumpkin leaves	102												
40	Barada Leaves	478												
41	Cauliflower leaves	68												
42	Sunusunia leaves													
43	Mint leaves	94												
44	Colocasia leaves	73												
45														
46														
47														
	**Roots and Tubers**	1004												
48	Carrot	118												
49	Colocasia	119												
50	Beetroot	117												
51	Onion big	122												
52	Potato	125												
53	Radish	129												
54	Sweet potato	130												
55	Orange flesh sweet potato													
56	Tapioca	131												
57	Yam ordinary	135												

Code for Col (4) – Frequency of Consumption
Daily=1; Twice /thrice a week=2; Once a week=3; Once in fifteen days=4; Once in a month=5; Occasionally=6
Code for Col (9), Col (11)
Daily=1; Twice /thrice a week=2; Once a week=3; Once in fifteen days=4; Once in a month=5; Occasionally=6

Code for Col(13)
Forest=1; Relatives/friends=2; Agriculture land=3; ICDS=4; Kind =5; Any other=6
* Total quantity collected during the last quarter

S.No	Food Group	Food Code	Frequency of consumption (Code)	Raw amounts consumed (g)		Purchased from PDS	Home grown		Purchased from market		Collected from other sources		Market Price (Rs./Kg)	Remarks
				Per HH/Day	Per CU/Day	Qty (Kg/Ltr)	Qty (Kg/Ltr)	Ref Per	Qty (Kg/Ltr)	Ref Per	Qty* (Kg/Ltr)	Other source code		
(1)	(2)	(3)	(4)	(5)	(6)	(7)	(8)	(9)	(10)	(11)	(12)	(13)	(14)	(15)
58	Yam Elephant	134												
59	Arrowroot	115												
60														
	**Other Vegetables**	1005												
61	Ash gourd	137												
62	Beans	138												
63	Broad beans	143												
64	Bottle gourd	141												
65	Bitter gourd	139												
66	Brinjal	142												
67	Cauliflower	144												
68	Cluster bean	146												
69	Colocasia stem	147												
70	Cow pea	148												
71	Drumstick	151												
72	French bean	155												
73	Jackfruit tender	158												
74	Ivy gourd	164												
75	Spine gourd/Kankoda	160												
76	Pumpkin	178												
77	Ridge gourd	180												
78	Ladies finger	166												

Code for Col (4) – Frequency of Consumption
Daily=1; Twice /thrice a week=2; Once a week=3; Once in fifteen days=4; Once in a month=5; Occasionally=6
Code for Col (9), Col (11)
Daily=1; Twice /thrice a week=2; Once a week=3; Once in fifteen days=4; Once in a month=5; Occasionally=6

Code for Col(13)
Forest=1; Relatives/friends=2; Agriculture land=3; ICDS=4; Kind =5; Any other=6
* Total quantity collected during the last quarter

S.No	Food Group	Food Code	Frequency of consumption (Code)	Raw amounts consumed (g) Per HH/Day	Raw amounts consumed (g) Per CU/Day	Purchased from PDS Qty (Kg/Ltr)	Home grown Qty (Kg/Ltr)	Home grown Ref Per	Purchased from market Qty (Kg/Ltr)	Purchased from market Ref Per	Collected from other sources Qty* (Kg/Ltr)	Collected from other sources Other source code	Market Price (Rs./Kg)	Remarks
(1)	(2)	(3)	(4)	(5)	(6)	(7)	(8)	(9)	(10)	(11)	(12)	(13)	(14)	(15)
79	Papaya green	172												
80	Parwar	173												
81	Plantain green	176												
82	Plantain stem	177												
83	Snake gourd	181												
84	Cucumber	149												
85														
86														
87														
88														
89														
	**Nuts and Oil seeds**	1006												
90	Coconut fresh	195												
91	Coconut dry	194												
92	Mustard seeds	206												
93	Groundnut fresh	202												
94	Groundnut boiled													
95	Groundnut roasted	203												
96	Niger seeds	207												
97	Sesamum (Til seed)	201												
98	Cashewnut fresh	192												
99	Cashewnut Roasted													

Code for Col (4) – Frequency of Consumption
Daily=1; Twice /thrice a week=2; Once a week=3; Once in fifteen days=4; Once in a month=5; Occasionally=6
Code for Col (9), Col (11)
Daily=1; Twice /thrice a week=2; Once a week=3; Once in fifteen days=4; Once in a month=5; Occasionally=6

Code for Col(13)
Forest=1; Relatives/friends=2; Agriculture land=3; ICDS=4; Kind =5; Any other=6
* Total quantity collected during the last quarter

S.No	Food Group	Food Code	Frequency of consumption (Code)	Raw amounts consumed (g)		Purchased from PDS	Home grown		Purchased from market		Collected from other sources		Market Price (Rs./Kg)	Remarks
				Per HH/Day	Per CU/Day	Qty (Kg/Ltr)	Qty (Kg/Ltr)	Ref Per	Qty (Kg/Ltr)	Ref Per	Qty* (Kg/Ltr)	Other source code		
(1)	(2)	(3)	(4)	(5)	(6)	(7)	(8)	(9)	(10)	(11)	(12)	(13)	(14)	(15)
100														
101														
	**Condiments and Spices**	1007												
102	Chillies dry	217												
103	Chillies green (fresh)	218												
104	Coriander seeds	221												
105	Cumin seeds	222												
106	Fenugreek seeds	223												
107	Garlic dry	224												
108	Ginger	225												
109	Pepper dry	232												
110	Tamarind	236												
111	Turmeric	237												
112														
113														
	**Fruits**	1008												
114	Amla	239												
115	Apple	240												
116	Bael	244												
117	Banana	245												
118	Cashew fruit	251												
119	Custard apple	304												

Code for Col (4) – Frequency of Consumption
Daily=1; Twice /thrice a week=2; Once a week=3; Once in fifteen days=4; Once in a month=5; Occasionally=6
Code for Col (9), Col (11)
Daily=1; Twice /thrice a week=2; Once a week=3; Once in fifteen days=4; Once in a month=5; Occasionally=6

Code for Col(13)
Forest=1; Relatives/friends=2; Agriculture land=3; ICDS=4; Kind =5; Any other=6
* Total quantity collected during the last quarter

S.No	Food Group	Food Code	Frequency of consumption (Code)	Raw amounts consumed (g)		Purchased from PDS	Home grown		Purchased from market		Collected from other sources		Market Price (Rs./Kg)	Remarks
				Per HH/Day	Per CU/Day	Qty (Kg/Ltr)	Qty (Kg/Ltr)	Ref Per	Qty (Kg/Ltr)	Ref Per	Qty* (Kg/Ltr)	Other source code		
(1)	(2)	(3)	(4)	(5)	(6)	(7)	(8)	(9)	(10)	(11)	(12)	(13)	(14)	(15)
120	Date palm	255												
121	Grapes	257												
122	Guava	261												
123	Jackfruit	264												
124	Jamun	266												
125	Lime	273												
126	Mango	278												
127	Orange	283												
128	Papaya	287												
129	Pineapple	294												
130	Tomato ripe	306												
131	Wood apple	309												
132	Zizypus	310												
133														
134														
135														
136														
137														
	**Fishes and Sea Foods**	1009												
138	Small fish													
139	Small fish (dry)													
140	Crabs													
141	Prawn (fresh)													

Code for Col (4) – Frequency of Consumption
Daily=1; Twice /thrice a week=2; Once a week=3; Once in fifteen days=4; Once in a month=5; Occasionally=6
Code for Col (9), Col (11)
Daily=1; Twice /thrice a week=2; Once a week=3; Once in fifteen days=4; Once in a month=5; Occasionally=6

Code for Col(13)
Forest=1; Relatives/friends=2; Agriculture land=3; ICDS=4; Kind =5; Any other=6
* Total quantity collected during the last quarter

S.No	Food Group	Food Code	Frequency of consumption (Code)	Raw amounts consumed (g)		Purchased from PDS	Home grown		Purchased from market		Collected from other sources		Market Price (Rs./Kg)	Remarks
				Per HH/Day	Per CU/Day	Qty (Kg/Ltr)	Qty (Kg/Ltr)	Ref Per	Qty (Kg/Ltr)	Ref Per	Qty* (Kg/Ltr)	Other source code		
(1)	(2)	(3)	(4)	(5)	(6)	(7)	(8)	(9)	(10)	(11)	(12)	(13)	(14)	(15)
142	Prawn (dry)													
143	Big Fish (Fresh)													
144	Big Fish (Dry)													
145														
146														
	**Meat and Poultry**	1010												
147	Egg duck													
148	Egg chicken													
149	Fowl (chicken)													
150	Goat meat													
151	Snail													
152	Beef													
153	Pork (pig meat)													
154														
155														
	**Milk and Milk products**	1011												
156	Milk Buffalo													
157	Milk Cow													
158	Milk Goat													
159	Curd													
160	Paneer													
161														
162														

Code for Col (4) – Frequency of Consumption
Daily=1; Twice /thrice a week=2; Once a week=3; Once in fifteen days=4; Once in a month=5; Occasionally=6
Code for Col (9), Col (11)
Daily=1; Twice /thrice a week=2; Once a week=3; Once in fifteen days=4; Once in a month=5; Occasionally=6

Code for Col(13)
Forest=1; Relatives/friends=2; Agriculture land=3; ICDS=4; Kind =5; Any other=6
* Total quantity collected during the last quarter

S.No	Food Group	Food Code	Frequency of consumption (Code)	Raw amounts consumed (g) Per HH/Day	Per CU/Day	Purchased from PDS Qty (Kg/Ltr)	Home grown Qty (Kg/Ltr)	Ref Per	Purchased from market Qty (Kg/Ltr)	Ref Per	Collected from other sources Qty* (Kg/Ltr)	Other source code	Market Price (Rs./Kg)	Remarks
(1)	(2)	(3)	(4)	(5)	(6)	(7)	(8)	(9)	(10)	(11)	(12)	(13)	(14)	(15)
	**Fats and Oils**	1012												
163	Butter													
164	Ghee cow													
165	Ghee Buffalo													
166	Vanaspathi/Dalda													
167	Cooking oil Specify :__________													
168														
	**Sugars**	1013												
169	Sugar													
170	Jaggery date palm													
171	Jaggery cane													
172	Sago													
173														
	**Beverages**	1014												
174														
175														
176														
	**Salt**	1015												
177	Salt crystal (Iodized)													
178	Salt crystal (Non Iodized)													
179	Salt Crystal Powder (Iodized)													
180	Salt Crystal Powder (Non Iodized)													

Code for Col (4) – Frequency of Consumption
Daily=1; Twice /thrice a week=2; Once a week=3; Once in fifteen days=4; Once in a month=5; Occasionally=6

Code for Col (9), Col (11)
Daily=1; Twice /thrice a week=2; Once a week=3; Once in fifteen days=4; Once in a month=5; Occasionally=6

Code for Col(13)
Forest=1; Relatives/friends=2; Agriculture land=3; ICDS=4; Kind =5; Any other=6
* Total quantity collected during the last quarter

S.No	Food Group	Food Code	Frequency of consumption (Code)	Raw amounts consumed (g) Per HH/Day	Per CU/Day	Purchased from PDS Qty (Kg/Ltr)	Home grown Qty (Kg/Ltr)	Ref Per	Purchased from market Qty (Kg/Ltr)	Ref Per	Collected from other sources Qty* (Kg/Ltr)	Other source code	Market Price (Rs./Kg)	Remarks
(1)	(2)	(3)	(4)	(5)	(6)	(7)	(8)	(9)	(10)	(11)	(12)	(13)	(14)	(15)
181	Salt Powder Free flowing (Iodized)													
182	Salt Powder Free flowing (Non Iodized)													
	**Wild Foods**	1016												
183														
184														
185														
186														
187														
188														
189														
190														
191														
	**Others**	1017												
192														
193														
194														
195														
196														
197														
198														
199														
200														

Code for Col (4) – Frequency of Consumption
Daily=1; Twice /thrice a week=2; Once a week=3; Once in fifteen days=4; Once in a month=5; Occasionally=6
Code for Col (9), Col (11)
Daily=1; Twice /thrice a week=2; Once a week=3; Once in fifteen days=4; Once in a month=5; Occasionally=6

Code for Col(13)
Forest=1; Relatives/friends=2; Agriculture land=3; ICDS=4; Kind =5; Any other=6
* Total quantity collected during the last quarter

Schedule 4: 24-hr Recall

Dist Name………………………. Dist Code__ __ __ Village Name……………….……. Village Code__ __ __ __ HH ID __ __ __ __ Date of Interview__ __ /__ __ /__ __

Individual ID					101	102	103	104	105	106	107	108	109	110		Remarks
Name																
Age																
Gender (M=1; F=2)																
Physiological Status																
Physical Activity																
Consumption Units																
Meal Pattern	Type of preparation	Food Stuff	Raw Amount (g)	Total Cooked Quantity	Individual Intake										Left Over	

Individual ID					101	102	103	104	105	106	107	108	109	110		Remarks
Meal Pattern	Type of preparation	Food Stuff	Raw Amount (g)	Total Cooked Quantity	Individual Intake										Left Over	

Food Compilation Sheet

Dist Name……………………….. Dist Code__ __ __ Village Name…………………..…. Village Code__ __ __ __ HH ID __ __ __ __ Date of Interview__ __ /__ __ /__ __

Individual ID				101	102	103	104	105	106	107	108	109	110	
Name														
Age														
Gender (M=1; F=2)														
Physiological Status														
Physical Activity														
Consumption Units														
Name of Food Stuff	Local Name	Food code	Total Qty (Raw)	Quantity of raw food stuff (gms/ml)										Left Over

Individual ID				101	102	103	104	105	106	107	108	109	110	
Name of Food Stuff	Local Name	Food code	Total Qty (Raw)	Quantity of raw food stuff (gms/ml)										Left Over
